# Genetic Elimination of Connective Tissue Growth Factor in the Forebrain Affects Subplate Neurons in the Cortex and Oligodendrocytes in the Underlying White Matter

**DOI:** 10.3389/fnana.2019.00016

**Published:** 2019-02-20

**Authors:** I-Shing Yu, Ho-Ching Chang, Ko-Chien Chen, Yi-Ling Lu, Horng-Tzer Shy, Chwen-Yu Chen, Kuang-Yung Lee, Li-Jen Lee

**Affiliations:** ^1^Laboratory Animal Center, College of Medicine, National Taiwan University, Taipei, Taiwan; ^2^Graduate Institute of Anatomy and Cell Biology, College of Medicine, National Taiwan University, Taipei, Taiwan; ^3^Department of Neurology, Chang Gung Memorial Hospital, Keelung, Taiwan; ^4^College of Medicine, Chang Gung University, Taoyuan, Taiwan; ^5^Institute of Brain and Mind Sciences, College of Medicine, National Taiwan University, Taipei, Taiwan; ^6^Neurobiology and Cognitive Science Center, National Taiwan University, Taipei, Taiwan

**Keywords:** CCN2, conditional knockout, subplate, cortical neuron, glial cells, dendrite, oligodendrocytes, myelin sheath

## Abstract

Connective tissue growth factor (CTGF) is a secreted extracellular matrix-associated protein, which play a role in regulating various cellular functions. Although the expression of CTGF has been reported in the cortical subplate, its function is still not clear. Thus, to explore the significance of CTGF in the brain, we created a forebrain-specific *Ctgf* knockout (Fb*Ctgf* KO) mouse model. By crossing *Ctgf*^fl/fl^ mice with *Emx1-Cre* transgenic mice, in which the expression of Cre is prenatally initiated, the full length *Ctgf* is removed in the forebrain structures. In young adult (2–3 months old) Fb*Ctgf* KO mice, subplate markers such as Nurr1 and Cplx3 are still expressed in the cortical layer VIb; however, the density of the subplate neurons is increased. Interestingly, in these mutants, we found a reduced structural complexity in the subplate neurons. The distribution patterns of neurons and glial cells, examined by immunohistochemistry, are comparable between genotypes in the somatosensory cortex. However, increased densities of mature oligodendrocytes, but not immature ones, were noticed in the external capsule underneath the cortical layer VIb in young adult Fb*Ctgf* KO mice. The features of myelinated axons in the external capsule were then examined using electron microscopy. Unexpectedly, the thickness of the myelin sheath was reduced in middle-aged (>12 months old), but not young adult Fb*Ctgf* KO mice. Our results suggest a secretory function of the subplate neurons, through the release of CTGF, which regulates the density and dendritic branching of subplate neurons as well as the maturation and function of nearby oligodendrocytes in the white matter.

## Introduction

Connective tissue growth factor (CTGF), also known as CCN family protein 2 (CCN2), is a 36–38 kDa cysteine-rich secreted protein. The CCN family members are characterized by four discrete functional domains: an insulin-like growth factor binding protein (IGFBP) module, a von Willebrand factor type C repeat (vWC) module, a thrombospondin type-1 repeat (TSP1) module and a cysteine-knot-containing (CT) module (Ramazani et al., 2018; Toda et al., 2018). Since these modules participate in a number of physiological processes such as growth factor binding and facilitate interactions between the extracellular matrix (ECM) and cell surface proteins, the CCN family members have been recognized as multi-functional matricellular organizers (Bornstein and Sage, 2002; Malik et al., 2015). CTGF also plays an important role during the development and regeneration of various connective tissues, such as bone (Canalis et al., 2010), cartilage (Takigawa, 2013), and blood vessels (Hall-Glenn et al., 2012). Genetic disruption of *Ctgf* in mice caused severe defects in various connective tissues and perinatal lethality (Ivkovic et al., 2003).

In fact, the expression of CTGF is not only restricted in the connective tissue but also in the forebrain regions including the olfactory bulb, endopiriform nucleus and the cortical subplate (Heuer et al., 2003). The cortical subplate lies directly underneath the cortical plate and contains the earliest generated neurons that play an important role in cortical development (Kostovic and Rakic, 1980; Chun and Shatz, 1989; Bayer and Altman, 1990; Price et al., 1997; McQuillen and Ferriero, 2005; Bystron et al., 2008; Judaš et al., 2010; Kanold and Luhmann, 2010; Hoerder-Suabedissen and Molnár, 2013; Hadders-Algra, 2018; Ohtaka-Maruyama et al., 2018).

To avoid the premature death in constitutive *Ctgf* knockouts and to investigate the function of CTGF *in vivo*, we generated a conditional *Ctgf* knockout (KO) mouse line, in which the CTGF protein expression is only eliminated in the excitatory neurons within the forebrain (Fb) structures. In the present study, we first confirmed the presence of cortical layer VIb (the preceding subplate zone) in Fb*Ctgf* KO mice and then examined the patterning of neurons and glial cells in the cortex of these mutants. The morphometric features of subplate neurons in the layer VIb was also characterized. Due to the anatomical proximity, we subsequently assessed the density of oligodendrocytes and ultra-structural features of myelinated axonal fibers in conditional knockout mice. Our results suggest that the subplate neuron-derived CTGF regulates the density and morphology of subplate neurons as well as the maturation and function of oligodendrocytes in the white matter.

## Materials and Methods

### Animals

Mice of the same genotype were group-housed (3–5) in the Laboratory Animal Center of the College of Medicine, National Taiwan University (AAALAC accredited), under a 12:12 light-dark cycle with free access to food and water. Except for the EM experiments, 2–3 month old young adult mice were used in this study. All animal handlings were in accordance with a protocol approved by the Institutional Animal Care and Use Committee of National Taiwan University. Efforts were constantly made to minimize animal discomfort as well as the number of mice used.

### Generation and Genotyping of Fb*Ctgf* KO Mice

Mouse genomic DNA encompassing *Ctgf* of 29.1 kb is acquired from the bacterial artificial chromosome RP24-346F6. The *Ctgf*^fl^ allele carrying *loxP*-flanked (floxed; fl) of *Ctgf* (exons 1–5) and a neomycin-resistance gene (*Neo*) flanked by two *FRT* sites, was introduced into mouse embryonic stem (ES) cells and the original *Ctgf* gene was replaced following homologous recombination. After a Southern blotting analysis, the targeted ES cells were injected into the C57BL/6J blastocyst and the resultant chimeras were mated with C57BL/6J females to obtain *Ctgf*^fl/fl^ mice of germ-line transmission.

*Emx1-IRES-Cre* knockin mice were purchased from the Jackson Laboratory (B6.129S2-*Emx1^tm1(cre)Krj^/J*) (stock No. 005628). Emx1-driven Cre recombinase is expressed in excitatory neurons within the forebrain structures including the olfactory bulb, neocortex, hippocampus, and the amygdala (Gorski et al., 2002; Chou et al., 2009). Following principle, DNA sequences flanked by two *loxP* sites were removed within the Cre-expressing cells (Sauer and Henderson, 1988). By crossing *Ctgf*^fl/fl^ mice with *Emx1-IRES-Cre* knockin mice, forebrain-specific *Ctgf* conditional knockout (Fb*Ctgf* KO) mice were generated.

For genotyping, tissues were obtained from mice at 7–14 days of age and digested with proteinase K (133 ng/ml) in lysis buffer (100 mM Tris–HCL, pH 8.8, 0.2% SDS, 200 mM NaCl, 1 mM KCl) overnight. The extracted DNA was then precipitated with isopropanol and re-suspended with 300 μl of TE buffer (10 mM Tris–HCL, 1 mM EDTA, pH 8.0). DNA samples tested for floxed *Ctgf* and function of Cre were put in an Emerald Amp master mix (Takara Bio Inc., Otsu, Japan) and then amplified by a T100 Thermal Cycler (Bio-Rad, CA, United States) for 35 cycles [for floxed *Ctgf* (CU and FD): 94°C for 10 min, 55°C for min, 72°C for 30 s; for function of Cre (CU and JD, GU and HD): 94°C for 10 min, 66°C for 1 min and 72°C for 30 s]. Primers used were CU: 5′-ATAGCGGC CGCAATACTTTTGACTTGCC-3′,FD: ATAGTCGACTGGCTTCCCAGTGTTTC T-3′, GU: 5′-ATAGCGGCCGCTCTGGTTCTGAACTCGAAAG-3′, HD: 5′-ATAGAATTCTTTTCTATATCA GGGTTC-3′, JD: 5′-ATAGTCGACTAGAAATACTTTTCTCATG-3′ (Figure 2).

In the present study, the mating pairs were *Emx1-IRES-Cre* mice carrying one allele of *Ctgf*^fl^ (*Emx1-Cre*; *Ctgf*^fl/+^). The offspring carrying *Emx1-IRES-Cre* and *Ctgf*^fl/fl^ were Fb*Ctgf* KO; whereas offspring carrying *Emx1-IRES-Cre* and *Ctgf^+/+^* were used as controls.

### Preparation of Tissue Sections

Control and Fb*Ctgf* KO mice were anesthetized with sodium pentobarbital (50 mg/kg, i.p.) and then perfused via the heart with phosphate-buffered saline (PBS), followed by 4% paraformaldehyde in 0.1 M PBS, pH 7.4. The brains were dissected and further fixed in the same fixative at 4°C overnight. Half brains were coronally sectioned at 30 μm thickness with a vibrating microtome (VT1000S, Leica Biosystems, Wetzlar, Germany) for immunohistochemistry. The remaining brain halves were used for Golgi-Cox impregnation.

### Immunohistochemistry

Thirty micrometer thick coronal sections were first incubated with 0.3% H_2_O_2_ in phosphate-buffered saline for 10 min to block endogenous peroxidase activity and then rinsed in PBS. Sections were then incubated with the blocking solution containing 4% normal goat serum, 1% bovine serum albumin and 0.4% Triton X-100 in PBS, for 2 h. The sections were then incubated with primary antibodies diluted with the blocking solution at room temperature overnight. The primary antibodies used were as follows: anti-CTGF (1:1000; Santa Cruz Biotechnology, Santa Cruz, CA, United States), anti-Complexin3 (Cplx3, 1:2000; Synaptic Systems, Göettingen, Germany), anti-Nurr1 (1:500; R&D Systems, Minneapolis, MN, United States), anti-NeuN (1:500; Merck Millipore, Darmstadt, Germany), anti-GAD67 (1:2000; Merck Millipore), anti-Iba1 (1:1000; GeneTex, Hsinchu, Taiwan, ROC), anti-S100β (1:500; Abcam, Cambridge, United Kingdom), anti-NG2 (1:400; Merck Millipore,), anti-MBP (1:4000; Covance, Princeton, NJ, United States) and anti-GST-pi (1:4000; BD Biosciences, San Jose, CA, United States). After incubation with the primary antibodies, sections were washed with PBS and then incubated with biotinylated secondary antibodies, against mouse IgG or rabbit IgG (1:500; The Jackson ImmunoResearch Laboratories, West Grove, PA, United States) for 2 h at room temperature. After the PBS washes, the sections were incubated with the reagents of the Vectastain (ABC kit, Vector Laboratories, Burlingame, CA, United States) for 1 h. Lastly, sections were reacted with 2 mg/ml of 3, 3′-diaminobenzidine (DAB) with 0.01% H_2_O_2_ in PBS, rinsed in PBS and mounted with a glycerol-based aqueous mounting medium on gelatin-coated slides and coverslipped.

### Immunofluorescence

Coronal sections of 30 μm-thick were transferred to the blocking solution as described above and then incubated with diluted primary antibodies, including anti-CTGF (1:1000; Santa Cruz) and anti-GM130 (1:500; BD Biosciences, Franklin Lake, NJ, United States) overnight at 4°C. After the PBS washes, sections were incubated with fluorophore-conjugated secondary antibodies (1:500, Jackson ImmunoResearch Laboratories) for 1 h at room temperature. Finally, sections were mounted in Fluoromount-G (plus DAPI, Southern Biotech, Birmingham, AL, United States).

### Measurement of Neurons and Glial Cells in the Cortex and Underlying White Matter

Images of immunostained coronal sections were taken from the somatosensory cortex. We measured the density of the subplate neurons by counting the numbers of Nurr1- and Cplx3-positive cells within a frame of 50 μm × 200 μm. Counting squares of 200 μm × 200 μm were used to estimate the density of immunopositive signals in the upper, middle and lower regions of the cortex, while a frame of 100 μm × 100 μm was used to count the cells in the external capsule (EC) and anterior commissure (AC). The thickness of the cortex was equally subdivided into 10 counting bins, starting from the pia surface to the border of the white matter. Due to the different densities of immunopositive signals, the width of the counting bin was set to 50 μm for NeuN-positive cells, 100 μm for Iba1 and S100β-positive cells and 150 μm for GAD67- and GST-pi-positive, 200 μm NG2-positive cells. The number of NeuN-, GAD67-, Iba1-, S100β-, NG2-, and GST-pi-positive cells within each bin were counted. The number of cells was totaled and the proportion of cells in each bin was calculated and represented as a percentage of the total number of cells across all 10 counting bins. In this counting system, bin 1 roughly corresponds to cortical layer I, bins 2–3 to layer II/III, bin 4 to layer IV, bins 5–7 to layer V, and bins 8–10 to layer VI, respectively.

### Golgi-Cox Impregnation and Morphometric Analysis of Neurons

The brain samples were kept in the impregnation solution from the FD rapid Golgi Stain kit (NeuroTechnologies, Ellicott City, MD, United States) at room temperature for 3 weeks. Impregnated samples were then washed with ddH_2_O and sectioned at a thickness of 100 μm using a vibratome (Leica). Sections were then incubated with a mixture of developer and fixer solutions provided in the same kit. Finally, the sections were washed and mounted on gelatin-coated slides.

From Golgi-Cox impregnated sections, subplate neurons were identified by their location. The dendritic morphology was examined under a light microscope (Olympus, Tokyo, Japan) with a 20×objective lens, captured with the Stereo Investigator system (MicroBrightField, Williston, VT, United States), reconstructed and analyzed with Neurolucida software (MicroBrightField). Neurolucida Explorer software was used to quantify the topological parameters including the number of primary dendrites, branching nodes, dendritic segments, and the terminal endings; as well as size-related parameters, such as dendritic length, intersections and the terminal endings at various distances from the soma (Ko et al., 2014).

### Transmission Electron Microscopy

Adult (3 months old) and middle-aged (>12 months old) male controls and Fb*Ctgf* KO mice were anesthetized with sodium pentobarbital (50 mg/kg, i.p.) and perfused with 0.1 M PB followed by 2% glutaraldehyde and 2% paraformaldehyde in 0.1M PB. Sections of 100 μm thickness were cut with a vibratome (Leica) and collected from Bregma 0.98 to 0.14 mm and post-fixed with 1% aqueous osmium tetroxide for 1 h. The white matter regions underneath the somatosensory cortex were taken for further processes. The samples were dehydrated in graded ethanol, washed with propylene oxide, and embedded in epoxy resin (Polysciences Inc., Warrington, PA, United States). Semi- and ultra-thin sections were cut perpendicularly to the axis of nerve fibers with a diamond knife on a Reichert-Jung Ultracut E ultramicrotome (Leica-Microsystems, Wetzlar, Germany). Ultra-thin sections were collected on copper grids (200 mesh, TAAB, Burks, United Kingdom) and stained with lead citrate and uranyl acetate. Photographs at a magnification of 10,000× were obtained using a transmission electron microscope (HITACHI H-7100, Tokyo, Japan) at 100 kV.

Twenty to thirty sections from each sample (in both genotypes, *n* = 3 mice in adult and four in middle-aged mice) were randomly taken and more than 450 myelinated fibers, with a complete myelin sheath, were collected from each group for morphological analysis. The inner axonal diameter (d) and outer myelin sheath diameter (D) of each fiber were measured by ImageJ software (NIH, Bethesda, MD, United States) and the g-ratio (d/D) was calculated. Data are displayed as a scatter-plot of g-ratio against the axon diameter.

### Data Analysis

Quantitative measurements of cell density, distribution and morphometric parameters were conducted blindly to the genotypes of the animals. All data are presented in the form of mean ± SEM. Statistical significance was analyzed by the Student’s *t*-test and an asterisks was used to indicate statistical significances (^∗^*p* < 0.05; ^∗∗^*p* < 0.01; ^∗∗∗^*p* < 0.001).

## Results

### CTGF Is Expressed in Cortical Layer VIb Subplate Neurons

Connective tissue growth factor expression has been reported in the cortical subplate (also recognized as layer VIb or layer VII) (Heuer et al., 2003; Wang et al., 2010; Montiel et al., 2011; Oeschger et al., 2012; Kondo et al., 2015; Hoerder-Suabedissen et al., 2018). The subplate zone is one of the earliest established structures that serves a significant role in consequential brain development (Kostovic and Rakic, 1980; Chun and Shatz, 1989; Bayer and Altman, 1990; Price et al., 1997; McQuillen and Ferriero, 2005; Bystron et al., 2008; Judaš et al., 2010; Kanold and Luhmann, 2010; Hoerder-Suabedissen and Molnár, 2013; Hadders-Algra, 2018; Ohtaka-Maruyama et al., 2018).

**FIGURE 1:**
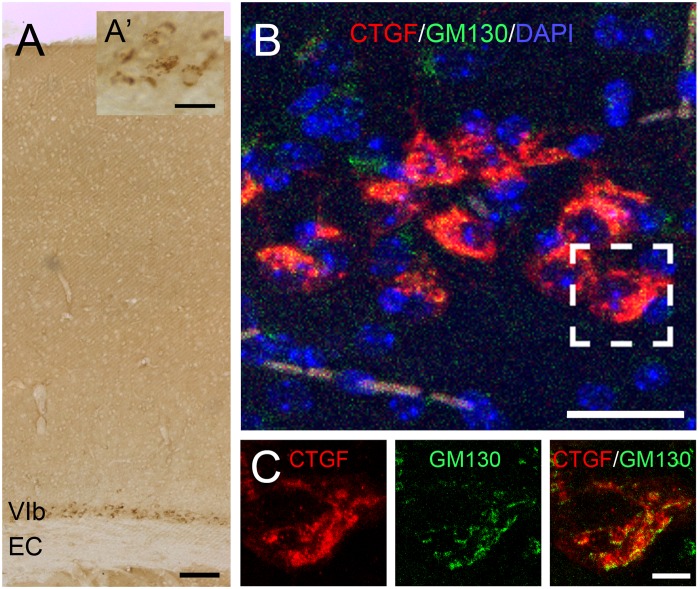
Expression of CTGF in the cerebral cortex. CTGF is expressed in the layer VIb of the cerebral cortex, above the external capsule (EC), in an adult mouse brain **(A)**. The CTGF-positive immunoreactive signals are located in the cytoplasm surrounding the nucleus **(A’)**. Intracellular distribution of CTGF is closely associated with the Golgi apparatus **(B,C)**. CTGF-positive signals (red) are close to signals of GM130, a *cis-*Golgi matrix protein (green) in the layer VIb neurons. Scale bar is 100 μm in A, 25 μm in **A’**, 25 μm in **B**, and 5 μm in **C**.

In the adult brain, although the subplate zone is no longer present, subplate neurons still persist as white matter interstitial cells in various mammalian species including cats and humans or as layer VIb neurons in many others including mice and rats (Chun and Shatz, 1989; Judaš et al., 2010; Marx et al., 2017; Hoerder-Suabedissen et al., 2018). Here, we used the term “subplate neurons” to describe those early developed neurons in the cortical layer VIb of adult mouse brains (Figure 1A). Within these cells, immunolabelled CTGF-positive puncta evidently surrounded the nucleus (Figure 1B). These signals are closely associated with the Golgi apparatus (Figure 1C), indicating a secretory function of the subplate neurons (Kondo et al., 2015).

### Generation of Forebrain-Specific *Ctgf* Knockout Mice

**FIGURE 2:**
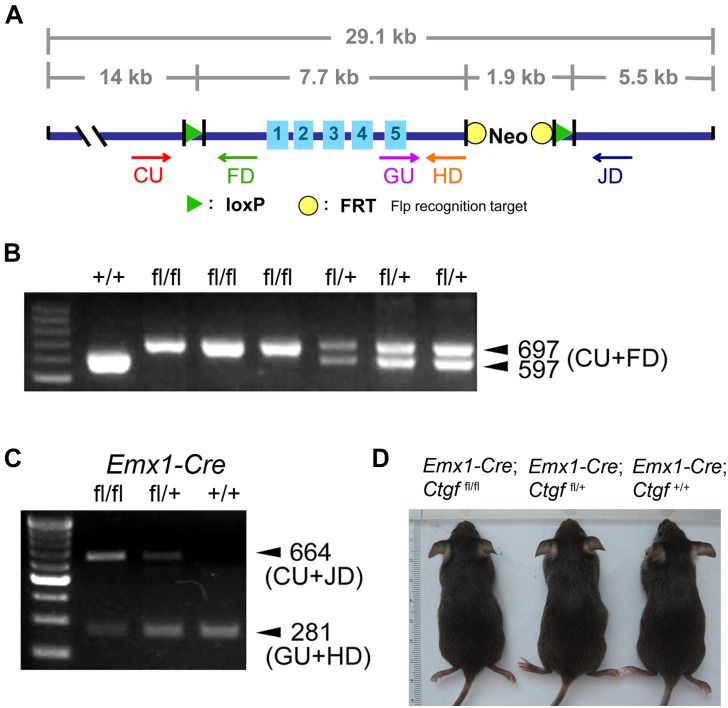
Generation and genotyping of forebrain-specific conditional *Ctgf* knockout mice. The strategy for generating *Ctgf* knockout (KO) mice **(A)**. A targeting vector carrying *loxP*-flanked *Ctgf* (exons 1–5) was designed employing a bacterial artificial chromosome contained mouse genomic DNA encompassing Ctgf of 29.1 kb. PCR products obtained from tail samples for distinguishing floxed (fl) *Ctgf* allele **(B)**. Genotyping of control (*Emx1-Cre*; *Ctgf*^+/+^), heterozygous (*Emx1-Cre*; *Ctgf*^fl/+^) and homozygous (*Emx1-Cre*; *Ctgf*^/fl/fl^) knockout mice using forebrain samples **(C)**. The outward appearances of control (*Emx1-Cre*; *Ctgf*^+/+^) and mutant (*Emx1-Cre*; *Ctgf*^fl/+^, *Emx1-Cre*; *Ctgf*^fl/fl^) mice **(D)**.

To elucidate the role of subplate neuron-derived CTGF in the brain, we generated a forebrain-specific *Ctgf* conditional knockout mouse model. A targeting vector carrying *loxP*-flanked (floxed; fl) *Ctgf* (exons 1–5) was designed (Figure 2A). A *Ctgf*^fl^ allele was established and *Ctgf*^fl/fl^ mice were then generated. The insertion of the *loxP* sequence was confirmed using PCR assays utilizing tail samples (Figure 2B). *Ctgf*^fl/fl^ mice were then crossed with *Emx1-IRES-Cre* knock-in mice to generate forebrain-specific *Ctgf* knockout (Fb*Ctgf* KO) mice. In Cre-expressing cells within the forebrain structure, including the cortical subplate, both *loxP*-flanked *Ctgf* alleles were removed. To verify the proper removal of *loxP*-flanked *Ctgf* alleles, we performed a PCR analysis employing the forebrain tissue. The amount of PCR product of *Ctgf* was largely reduced in homozygous KO (*Emx1-Cre*; *Ctgf*^fl/fl^) mice, relative to that in heterozygous KO (*Emx1-Cre*; *Ctgf*^fl/+^) and control (*Emx1-Cre*; *Ctgf*^+/+^) mice (Figure 2C). However, there was still a trace of the *Ctgf* PCR product in the homozygous KO group which could be attributed by the *Ctgf* gene in inhibitory neurons which escaped from *Emx1-*Cre-mediated gene deletion (Gorski et al., 2002). In this study, we used *Emx1-Cre*; *Ctgf*^fl/fl^ mice as Fb*Ctgf* KOs and *Emx1-Cre*; *Ctgf*^+/+^ mice as controls, respectively. Fb*Ctgf* KO mice were viable and fertile. No significant general difference in external appearance among Fb*Ctgf* KO, heterozygous and control mice was noted (Figure 2D).

### Expression of Subplate Markers in Cortical Layer VIb

**FIGURE 3:**
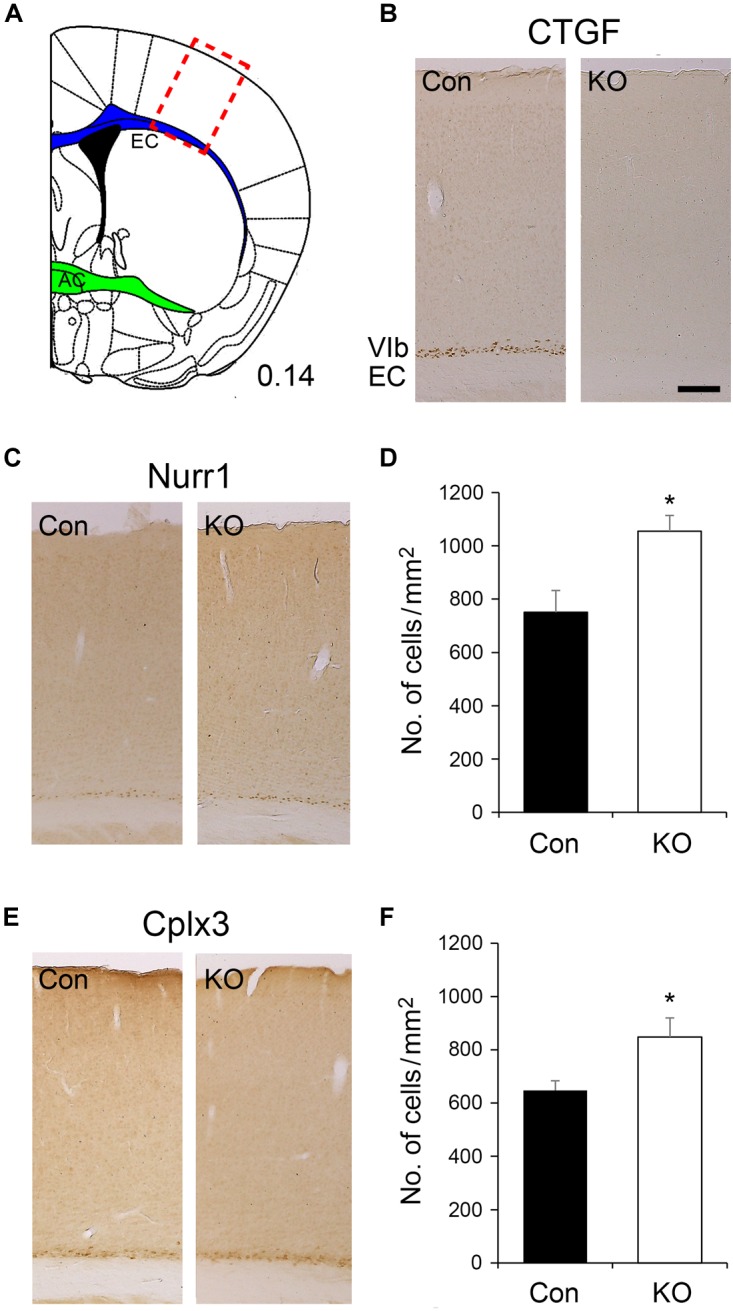
Expression of subplate neuron markers in the layer VIb of the somatosensory cortex. The somatosensory cortex is labeled (red rectangle) in a brain section in which the external capsule (EC) is marked in blue while the anterior commissure (AC) is marked in green **(A)**. CTGF is expressed in the layer VIb neurons of control (Con) mice but not in forebrain-specific *Ctgf* knockout (KO) mice **(B)**. Nurr1 is expressed in layer VIb neurons of both controls and mutants **(C)**. A significantly higher density of Nurr1-positive cells was identified in Fb*Ctgf* KO (*n* = 5) mice, compared with control (*n* = 5) mice **(D)**. Cplx3 is a subplate neuron marker which is expressed in the layer VIb neurons of both control and Fb*Ctgf* KO mice **(E)**. Similar to Nurr1 immunostaining, the density of Cplx3-positive cells in Fb*Ctgf* KO (*n* = 5) mice was higher than in controls (*n* = 5) **(F)**. Scale bar is 200 μm. Results are mean ± SEM (^∗^*p* < 0.05).

The expression of *Ctgf* mRNA starts with a low level at embryonic day (E) 14 (Heuer et al., 2003) while the *Emx1*-driven Cre recombinase is turned on as early as E10.5 in mice (Gorski et al., 2002). In Fb*Ctgf* KO mice, the full-length *Ctgf* gene was thus eliminated in the forebrain excitatory neurons before its native expression. Since the birth date of the subplate neurons is around E11.5 and E12.5 in mice (Hoerder-Suabedissen and Molnár, 2012), removal of *Ctgf* may affect the development of the subplate neurons. To evaluate the CTGF loss of function effect on subplate neurons, markers such as CTGF, Nurr1, and Cplx3 (Montiel et al., 2011) were examined in the somatosensory cortex (Figure 3). In adult control mice, CTGF was expressed in layer VIb (the preceding subplate zone) situated above the underlying white matter, external capsule (EC); while CTGF was not detectable in Fb*Ctgf* KO mice (Figure 3B). This result confirmed the successful removal of *Ctgf* in the forebrain and indicates that CTGF is expressed solely in the excitatory neurons. Despite the absence of CTGF in layer VIb of Fb*Ctgf* KO mice, Nurr1, and Cplx3 expressions were still positively displayed in the subplate neurons in both genotypes (Figure 3C–E). Notably, the densities of both Nurr1- and Cplx3-positive neurons in layer VIb were higher in the KO, compared to the control mice (Figure 3D–F). Since the thicknesses of both the cortex and layer VIb are comparable between the control and Fb*Ctgf* KO mice, the higher subplate neuron density represents the increase of the subplate neurons in the KO mice. This result suggests an autocrine or paracrine function of the subplate neuron-derived CTGF in regulating local neuron density.

### Morphology of Subplate Neurons in Cortical Layer VIb

**FIGURE 4:**
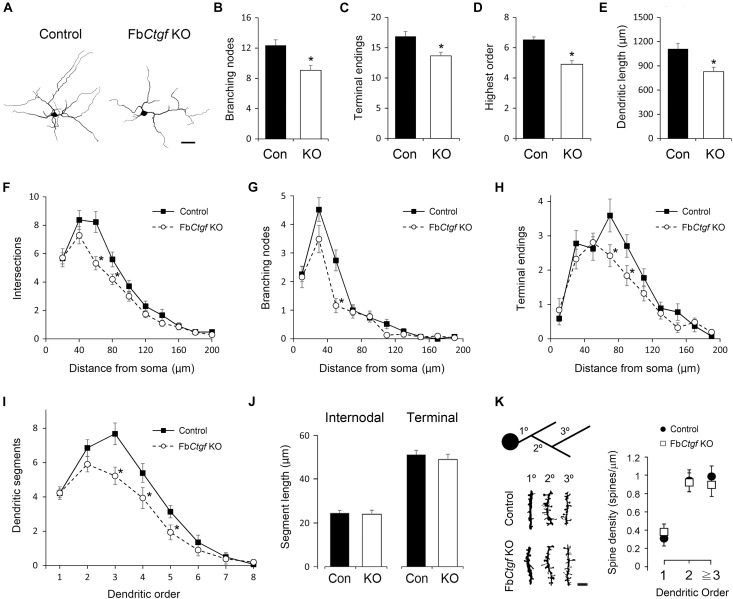
Morphometric analysis of layer VIb neurons in the somatosensory cortex. Golgi-Cox impregnated layer VIb neurons were collected (40 neurons from 6 control mice and 42 neurons from Fb*Ctgf* KO mice, respectively) and reconstructed. Scale bar is 20 μm **(A)**. The dendritic features including the numbers of branching nodes **(B)** and terminals endings **(C)**, highest order **(D)**, and total dendritic length **(E)** were measured. The complexity and branching pattern of dendrites were estimated using the concentric sphere method **(F–H)** and the number of dendritic segments **(I)**. The lengths of intermodal segments and terminal segments were also measured **(J)**. Dendritic segments of different orders were obtained and the densities of dendritic spines were quantified. Scale bar is 5 μm **(K)**. Results are means ± SEM (^∗^*p* < 0.05).

We next examined the morphometric features of subplate neurons within the cortical layer VIb in adult mice. Neurons were collected from Golgi-Cox-impregnated samples and 3D-reconstructed (Figure 4A). The number of bifurcation nodes (Figure 4B), terminal endings (Figure 4C) as well as the highest order (Figure 4D) were decreased in the subplate neurons of Fb*Ctgf* KO mice, compared to the control mice. In addition, the total dendritic length was also reduced in mutant mice (Figure 4E). We then examined the complexity of dendrites using the concentric ring method of Sholl (Wang et al., 2012). The center of the concentric rings of different radii was placed on the soma and the intersections between the dendrites and concentric rings were measured (Figure 4F). The numbers and locations of bifurcation nodes (Figure 4G) and terminal endings (Figure 4H) were also plotted. Compared with the controls, the subplate neurons from Fb*Ctgf* KO mice had fewer intersections, bifurcation nodes and terminal endings in the regions around 40–80 μm from the soma, suggesting branching deficiencies in these neurons. Next, we measured the number of segments in each dendritic order. The number of primary (first order) dendrites were comparable between genotypes (Figure 4I), indicating that the initial protrusion of neurites is not affected by CTGF removal. Instead, the number of segments declined dramatically from the third order in neurons of mutant mice, compared with the control (Figure 4I). These results indicate the branching defects in the subplate neurons in Fb*Ctgf* KO mice. The length of dendritic segments was also measured. The length of internodal and terminal segments was comparable between these two groups (Figure 4J), suggesting that the elongation process of the subplate neuronal dendrites was not affected in the absence of CTGF. To measure the density of dendritic spines in the subplate neurons, the dendritic spines of Golgi-Cox-impregnated subplate neurons were collected from different orders. There was no significant difference in spine density between the control and mutant groups (Figure 4K).

### Patterning of Cortical Neurons

**FIGURE 5:**
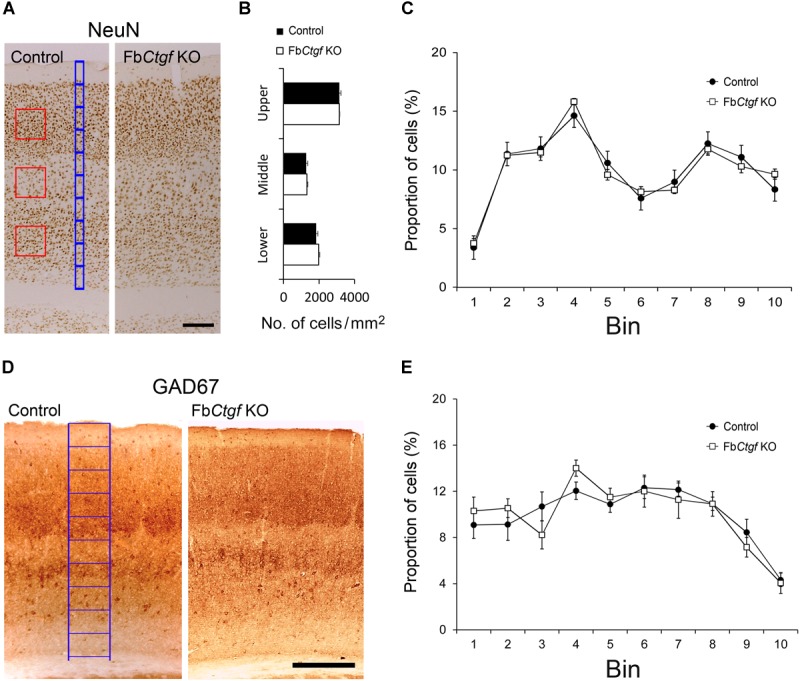
Density and distribution of neurons in the somatosensory cortex. Neurons were labeled with pan-neuronal markers, NeuN **(A)**. Counting squares of 200 μm × 200 μm (red squares) were given to the upper (layers II–IV), middle (layer V), and lower (layer VIa) regions of the cortex. The densities of NeuN-positive neurons in the cortex were estimated in control and Fb*Ctgf* KO mice **(B)**. The distribution of cortical neurons was measured using 10 counting bins of 50 μm in width (blue bins) from the pia surface to the edge of the white matter. In this system, from the top, bin 1 roughly corresponds to cortical layer I, bins 2–3 to layer II/III, bin 4 to layer IV, bins 5–7 to layer V, and bins 8–10 to layer VI, respectively. The distributions of NeuN-positive neurons were similar between control and Fb*Ctgf* KO mice **(C)**. Inhibitory neurons in the somatosensory cortex were revealed with GAD67 immunostaining **(D)**. The distribution of inhibitory cortical neurons was measured using 10 counting bins of 150 μm in width (blue bins) from the pia surface to the edge of white matter. The distributions of GAD67-positive neurons were similar between control and Fb*Ctgf* KO mice **(E)**. Scale bars are 200 μm. *n* = 5 in each genotype. Results are means ± SEM.

The migration of neocortical projection neurons initiates at E11.5 and further contributes to the establishment of the six-layered neocortex (Kwan et al., 2012). A recent study has demonstrated an important role of subplate neurons in radial migration of cortical neurons (Ohtaka-Maruyama et al., 2018). We wondered whether the absence of the subplate neuron-derived CTGF would affect the neural patterning of the cortex. Neurons in the somatosensory cortex were labeled with a pan neuronal marker, NeuN (Figure 5A), and quantified. The density of NeuN-positive cells in upper, middle and lower cortical regions were comparable between control and Fb*Ctgf* KO mice (Figure 5B). To measure the distribution of cortical neurons, the thickness of the cortex was equally divided into 10 counting bins with a width of 50 μm. In this measurement, bin 1 is roughly equivalent to the layer I; bins 2–3 to layer II/III; bin 4 to layer IV; bins 5–7 to layer V, and bins 8–10 to layer VI, respectively (Figure 5A). The cortex thicknesses were comparable between the controls and Kos. This analysis showed that the distributions of NeuN-positive cells in each counting bin were similar between control and Fb*Ctgf* KO mice (Figure 5C). The expression of the GABAergic neuronal marker GAD67, was also unchanged between control and Fb*Ctgf* KO mice (Figure 5D,E). These results indicated that the patterning of cortical neurons was not affected by the removal of forebrain CTGF.

### Distribution of Astrocytes and Microglia in the Cortex and White Matter

**FIGURE 6:**
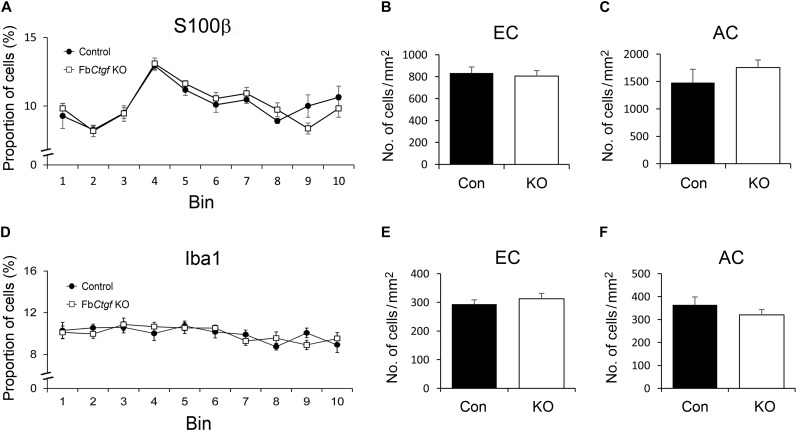
Distributions of astrocytes and microglia in the brain. In the somatosensory cortex, the distribution of S100b-positive astrocytes and Iba1-positive microglia was measured using 10 counting bins of 100 μm from the pia surface to the edge of the white matter. In this counting system, the distributions of astrocytes were similar between control and Fb*Ctgf* KO mice **(A)**. The densities of astrocytes were comparable between genotypes in both EC **(B)** and AC **(C)**. Iba1-positive microglia were evenly distributed in the cortex of both genotypes **(D)**. The densities of microglia were similar between control and Fb*Ctgf* KO mice in both EC **(E)** and AC **(F)**. *n* = 5 in each genotype. Results are means ± SEM.

Although the expression of CTGF is excitatory neuron-specific, the recipients might include other cell types. We therefore examined the pattern of a variety of glial cells in the gray and white matter. We first assessed the distribution and density of astrocytes by employing an antibody against S100β (Supplementary Figure S1). The S100β-positive astrocytes were scattered across the cortical layers (Supplementary Figure S1A) and demonstrated no significant difference in patterning between controls and mutants (Figure 6A). The densities of S100β-positive astrocytes were also examined in the white matter in two different locations; the external capsule (EC) underlying the somatosensory cortex and the anterior commissure (AC) in the basal forebrain (Supplementary Figure S1B). While the EC is close to the CTGF-releasing subplate neurons, the AC is located at a distance away from any CTGF-producing sources (Figure 3A). However, the densities of astrocytes were not similar between control and Fb*Ctgf* KO mice, in neither of the locations (Figure 6B,C). Next, the density and distribution of microglia was examined using Iba1 immunohistochemistry (Supplementary Figure S2B). Our results showed that Iba1-positive microglia were distributed evenly in the cerebral cortex in both control and Fb*Ctgf* KO mice (Supplementary Figure S2A) and were comparable between the two mouse groups (Figure 6D). The density of Iba1-positive microglia in the EC and AC were also indistinguishable between control and Fb*Ctgf* KO mice (Figure 6E,F and Supplementary Figure S2B). Altogether, our results indicated that the densities and distributions of astrocytes and microglia were not affected by the absence of the subplate neuron-derived CTGF.

### Distribution of Oligodendrocytes in the Cortex and White Matter

**FIGURE 7:**
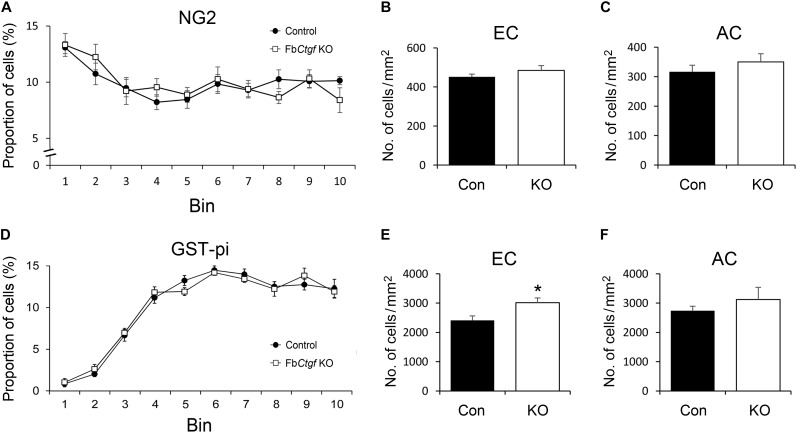
Distribution of oligodendrocytes in the brain. In the somatosensory cortex, higher proportions of NG2-positive oligodendrocytes were distributed in the upper layers of the cortex relative to the middle and lower regions in both control and Fb*Ctgf* KO mice **(A)**. The densities of NG2-positive oligodendrocytes were similar between genotypes in both EC **(B)** and AC **(C)**. Notably, fewer GST-pi-positive oligodendrocytes were counted in the upper layers of the somatosensory cortex in both control and Fb*Ctgf* KO mice **(D)**. In the EC, the density of GST-pi-positive oligodendrocytes was higher in Fb*Ctgf* KO mice than in control mice **(E)**. In the AC, the densities of GST-pi-positive oligodendrocytes were comparable between genotypes **(F)** *n* = 5 in each genotype. Results are means ± SEM (^∗^*p* < 0.05).

Previous studies have demonstrated the role of CTGF in regulating the differentiation of oligodendrocytes (Stritt et al., 2009; Lamond and Barnett, 2013; Ercan et al., 2017). The pattern of immature and mature oligodendrocytes were therefore examined in our mouse model. NG2-positive immature oligodendrocytes were distributed in the cortex with greater density in the superficial regions and relatively even in the whitematter (Supplementary Figure S3). The pattern and density of NG2-positive cells were comparable between control andFb*Ctgf* KO mice (Figure 7A–C). In contrast to the pattern of NG2-positive immature oligodendrocytes, less GST-pi-positive mature oligodendrocytes were distributed in the superficial regions of the cortex (Figure 7D and Supplementary Figure S4A). Notably, in the EC, the density of GST-pi-positive mature oligodendrocytes was increased in Fb*Ctgf* KO mice compared to control mice (Figure 7E and Supplementary Figure S4B); whereas in the AC, the densities were quite similar (Figure 7F). These results suggested a paracrine function of the subplate neuron-secreted CTGF which impedes the maturation of oligodendrocytes in the proximatewhite matter.

### Myelinated Axons in the External Capsule

**FIGURE 8:**
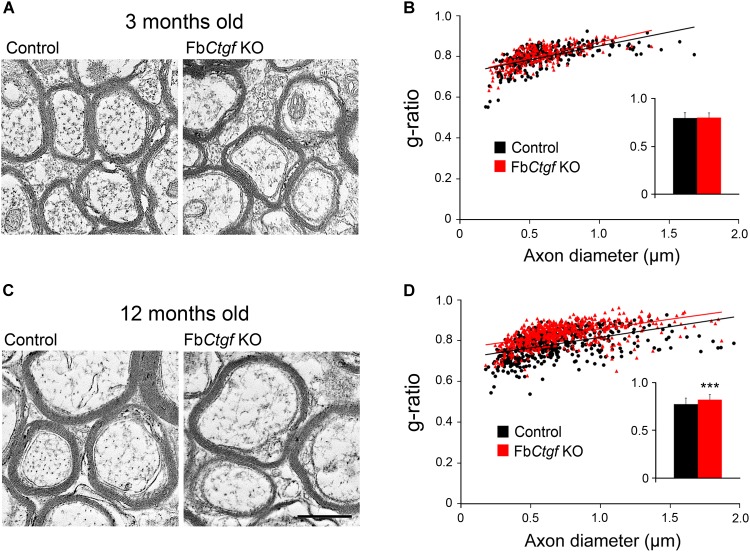
Myelinated axons in the external capsule. Photomicrographs were taken from the external capsule underneath the somatosensory cortex of the young adult **(A)** and middle-aged **(B)** mice using a transmission electron microscope. The inner axonal diameter and outer myelin sheath diameter of each fiber were measured, and the g-ratio was determined (*n* > 450 myelinated fibers from three mice per genotype in the young adult group and four mice per genotype in the middle-aged group). In the young adult group, no significant change in g-ratio was found between genotypes **(C)**. In the middle-aged group, a higher g-ratio was noted in Fb*Ctgf* KO mice relative to the age-matched control mice **(D)**. Scale bar is 500 nm. Results are means ± SEM (^∗∗∗^*p* < 0.001).

Oligodendrocytes support and provide insulation to axons by producing myelin sheaths that wrap individual axonal fibers. Since the density of mature oligodendrocyte is increased in the EC of adult Fb*Ctgf* KO mice, we wondered if the function of oligodendrocytes is affected by the removal of CTGF. Therefore, the morphometric features of myelin sheaths in the EC of control and Fb*Ctgf* KO mice were checked using transmission electron microscopy (Figure 8). We measured the inner axonal diameter (d) and outer myelin sheath diameter (D) of each myelinated axonal fiber and the g-ratio (d/D) was calculated. The g-ratio of myelinated axons in the EC of young adult (3 months old) mice (Figure 8A) displayed no significant difference between control and Fb*Ctgf* KO mice (Figure 8B). On the other hand, we also collected samples from middle-aged (>12 months old) mice (Figure 8C) and found a greater g-ratio in KO mice compared to the age-matched controls (Figure 8D), indicating a thinner myelin sheath in the EC of middle-aged Fb*Ctgf* KO mice. These results suggested that the subplate neuron-derived CTGF could regulate the maturation and function of oligodendrocytes in the white matter in a paracrine manner.

## Discussion

Connective tissue growth factor is a secreted matricellular protein expressed in specific brain regions, including the cortical subplate or layer VIb. In this study, we generated a conditional *Ctgf* KO mouse model to study the function of subplate neuron-derived CTGF in the brain. While the cortical subplate is still present as the layer VIb in adult Fb*Ctgf* KO mice, the density and morphology of subplate neurons were altered in these mice, indicating an autocrine/paracrine function of the subplate neuron-derived CTGF acting on the subplate neurons. In the white mater, the density of mature oligodendrocyte and the g-ratio of the myelin sheath increased in the EC of the mutants, indicating a paracrine function of the subplate neuron-derived CTGF acting on the nearby oligodendrocytes. Collectively, our findings suggested a secretory function of the cortical subplate neurons in the brain.

### Subplate Neuron-Derived CTGF Modulates the Density and Morphology of Subplate Neurons

The neurogenesis of subplate neurons takes place at E11.5 to E12.5 in mice (Hoerder-Suabedissen and Molnár, 2012) in the ventricular zone and these neurons then migrate radially. The subplate neuron-derived CTGF starts to express around E14 (Heuer et al., 2003), which may modulate the developing subplate neurons. In KO mice, we observed increased numbers of Nurr1- and Cplx3-positive neurons in the layer VIb compared to the control mice. Nurr1 and Cplx3 are well-accepted makers for subplate neurons (Montiel et al., 2011) and these findings suggest that the subplate neuron-derived CTGF may negatively regulate the survival of neurons in this restricted region, but not other cortical layers. In fact, CTGF was shown to be expressed in the olfactory bulb (particularly in the external tufted cells) (Heuer et al., 2003) and suppressed the survival of periglomerular inhibitory interneurons through activating proapoptotic TGF-β2 signaling (Khodosevich et al., 2013). Whether CTGF in the cortical subplate also mediates proapoptotic effects remains to be determined.

On the other hand, the dendritic morphology of subplate neurons is also affected in these mutants. Dendritic growth is achieved largely by dendritic branching and elongation (Lee et al., 2005). In the subplate neurons of Fb*Ctgf* KO mice, the dendritic complexity is reduced, yet the lengths of intermodal and terminal segments are not changed. These results suggest a unique role of CTGF in regulating dendritic branching, regardless of its inhibitory role in the subplate neuronal number. In fact, another CCN family protein Cyr61, also known as CCN1, has been shown to promote dendritic arborization in cultured hippocampal neurons. While Cyr61 knockdown using shRNA simplifies the dendrites, overexpression of Cyr61 increases the dendritic trees (Malik et al., 2013). The insulin-like growth factor binding protein (IGFBP) module is a common domain shared in all CCN family members (Ramazani et al., 2018; Toda et al., 2018). IGF-1 is expressed in the cortical neurons and play an important role in the branching of dendrites (Cheng et al., 2003). Removal of CTGF in the cortical subplate might therefore influence the dendritic branching in the subplate neurons. To our knowledge, our finding is the first evidence which indicates that CCN family members enhance dendritic arborization *in vivo*.

### Subplate Neuron-Derived CTGF in the Patterning of Cortical Neurons

A recent study has demonstrated that the synaptic transmission of the subplate neurons control the radial migration of cortical neurons during early cortical development (Ohtaka-Maruyama et al., 2018). Since reduced dendritic branches of subplate neurons were observed in Fb*Ctgf* KO mice and may potentially cause a defect in synaptic transmission (Liao and Lee, 2012) and neuronal migration, cortical patterning in these mutants were carefully evaluated. Unexpectedly, the density and distribution of NeuN- and GAD67-positive cells were not changed in Fb*Ctgf* KO mice, which argued against the possibility. In addition, neither of the analyses on astrocytes and microglia showed differences in these mutant mice. One possibility is that the developmental irregularities may have occurred at earlier time points, which we were unable to identify. Further studies focusing on cortical development during embryonic and neonatal stages may be necessary.

### CTGF Regulates the Maturation and Function of Oligodendrocytes

The vWC, TSP1, and CT modules of the CTGF protein interact with a great spectrum of proteins (Toda et al., 2018). Among these CTGF-binding proteins, integrins, low-density lipoprotein receptor-related protein 1 (LRP1), and vascular endothelial growth factor (VEGF) are known to modulate the development of oligodendrocyte in various aspects (Wu and Reddy, 2012; Auderset et al., 2016; Safina et al., 2016). For example, αvβ3 integrin induces the proliferation of oligodendrocyte precursor cells (Baron et al., 2002); while αvβ5 integrin and VEGFA are involved in the migration of oligodendrocyte precursors (Gudz et al., 2006; Hayakawa et al., 2011). CTGF has been shown to suppress the differentiation of oligodendrocytes in different models. However, the detailed mechanisms are still not clear. An *in vivo* study utilizing exogenous administration of CTGF in the ventricles of neonatal rodent brains, caused a reduction of mature oligodendrocytes (Stritt et al., 2009). Another *in vitro* study showed an inhibitory effect CTGF in oligodendrocyte differentiation using purified rat oligodendrocyte precursor cells (Lamond and Barnett, 2013). Oligodendrocyte precursor cells could continually proliferate and differentiate into oligodendrocytes in an adult central nervous system (Young et al., 2013; Fernandez-Castaneda and Gaultier, 2016). In adult brains, we observed an increased number of GST-pi-positive mature oligodendrocytes in the EC of Kos, supporting the notion that CTGF may inhibit maturation of oligodendrocytes located directly underneath the subplate neurons (Stritt et al., 2009; Lamond and Barnett, 2013). In the AC, which is 2.5 mm away from the subplate neurons and contains axon fibers derived from subplate neurons (Jacobs et al., 2007), the number of mature oligodendrocyte remained unchanged between KO and control mice. These findings may suggest that CTGF is secreted from the soma or dendrites of subplate neurons and suppresses the maturation of oligodendrocytes in a paracrine manner. On the other hand, our results showed no differences of NG2-positive immature oligodendrocytes in the white matter between the controls and mutant mice, which implies that CTGF may not be essential for the proliferation or early development of oligodendrocytes. Nevertheless, the details of how CTGF modulates oligodendrocyte development at different stages warrant further investigation.

The inhibitory effect of CTGF on oligodendrocyte maturation and myelination has been reported in neuron-specific *Ctgf* KO mice (Ercan et al., 2017). In this model, the intensity of myelin basic protein-positive signals and the percentage of myelinated axons are increased in the cortex and EC at P21, while the thicknesses of the myelin sheaths were not changed in mutants. In our Fb*Ctgf* KO mice, the thickness of the myelin sheath is comparable to the control mice at 3 months old. However, we found that a reduction of the myelin sheath thickness occurred in middle-aged but not young KO mice, which is quite different from what we saw in the older population. During normal aging, the thickness of the myelin sheath usually increases with age (Peters, 2002; Xie et al., 2014; Li et al., 2017). In middle-aged Fb*Ctgf* KO mice, the reduction of the myelin thickness was evident and the underlying mechanism requires further investigation.

A thinner myelin sheath might be a sign of remyelination (Xie et al., 2014). In this regard, CTGF might be a suppressive signal detrimental for myelin regeneration. To clear this issue, a “cuprizone model” of demyelination and remyelination (Skripuletz et al., 2011; Gudi et al., 2014) may be worth conducting. Cuprizone administration induces demyelination by reducing the number of mature oligodendrocytes and these effects can be reversed (maturation of oligodendrocytes and formation of the myelin sheath) after the cuprizone treatment is ceased. The role(s) of CTGF and associated ECM proteins in oligodendrocyte differentiation and myelin sheath formation could also be further analyzed (Lau et al., 2013; Wheeler and Fuss, 2016; Dombrowski et al., 2017; Franklin and Ffrench-Constant, 2017; Pu et al., 2018).

### Secretory Function of Subplate Neurons

A large amount of rough endoplasmic reticulum (rER) and high ER stress have been noted in the subplate neurons of neonatal (P8) rats, and these findings proposed that the subplate neurons are active protein-secreting neurons during early development (Kondo et al., 2015). In this study, we showed intracellular distribution of CTGF in normal mice and altered morphology and density of the subplate neurons in Fb*Ctgf* KO mice, supporting a secretory function of the subplate neurons. We also exhibited increased mature oligodendrocytes and a reduction in myelin sheath thickness in the EC of young adult and middle-aged Fb*Ctgf* KO mice, respectively. These findings signified for the first time, that the paracrine function in the subplate neurons continue beyond the developmental period and persist in adult and even aging brains. To support this notion, an immunoEM technique, focusing on the intracellular distribution of CTGF in the subplate neurons acquired from brains of different age groups, may be worth conducting.

The role of subplate neurons in the developing cortex have been extensively studied. However, since subplate neurons still persist in adult brains, hypotheses stating a shift in function during their lifetime have been proposed (Woo et al., 1991; Torres-Reveron and Friedlander, 2007; Liao and Lee, 2012). Our results suggested a secretory function of the subplate neurons, through the release of CTGF, that modulate the survival and morphology of the subplate neurons during cortical development and the maturation and function of oligodendrocytes throughout their lifetime.

## Author Contributions

I-SY, H-CC, K-CC, Y-LL, H-TS, and C-YC conducted the experiments, analyzed the data, and composed the manuscript draft. K-YL and L-JL prepared the manuscript.

## Conflict of Interest Statement

The authors declare that the research was conducted in the absence of any commercial or financial relationships that could be construed as a potential conflict of interest.
