# Supplementary figures and images for: Genetic Elimination of Connective Tissue Growth Factor in the Forebrain Affects Subplate Neurons in the Cortex and Oligodendrocytes in the Underlying White Matter
Source: Front Neuroanat. 2019 Feb 20;13:16. doi: 10.3389/fnana.2019.00016 (PMC6391576; doi:10.3389/fnana.2019.00016)

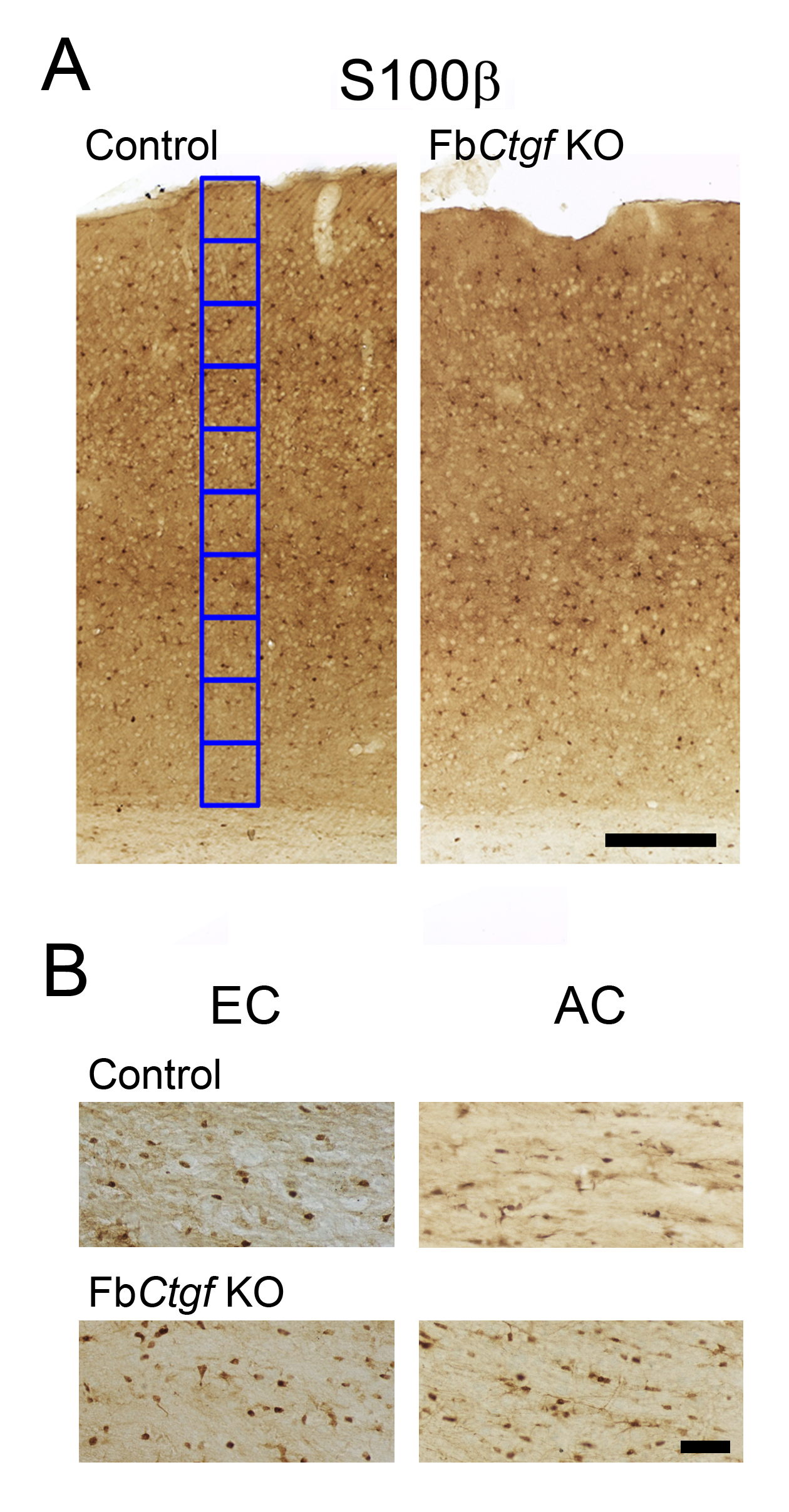

Supplement: Supplementary file 1 [file Image_1.TIF]

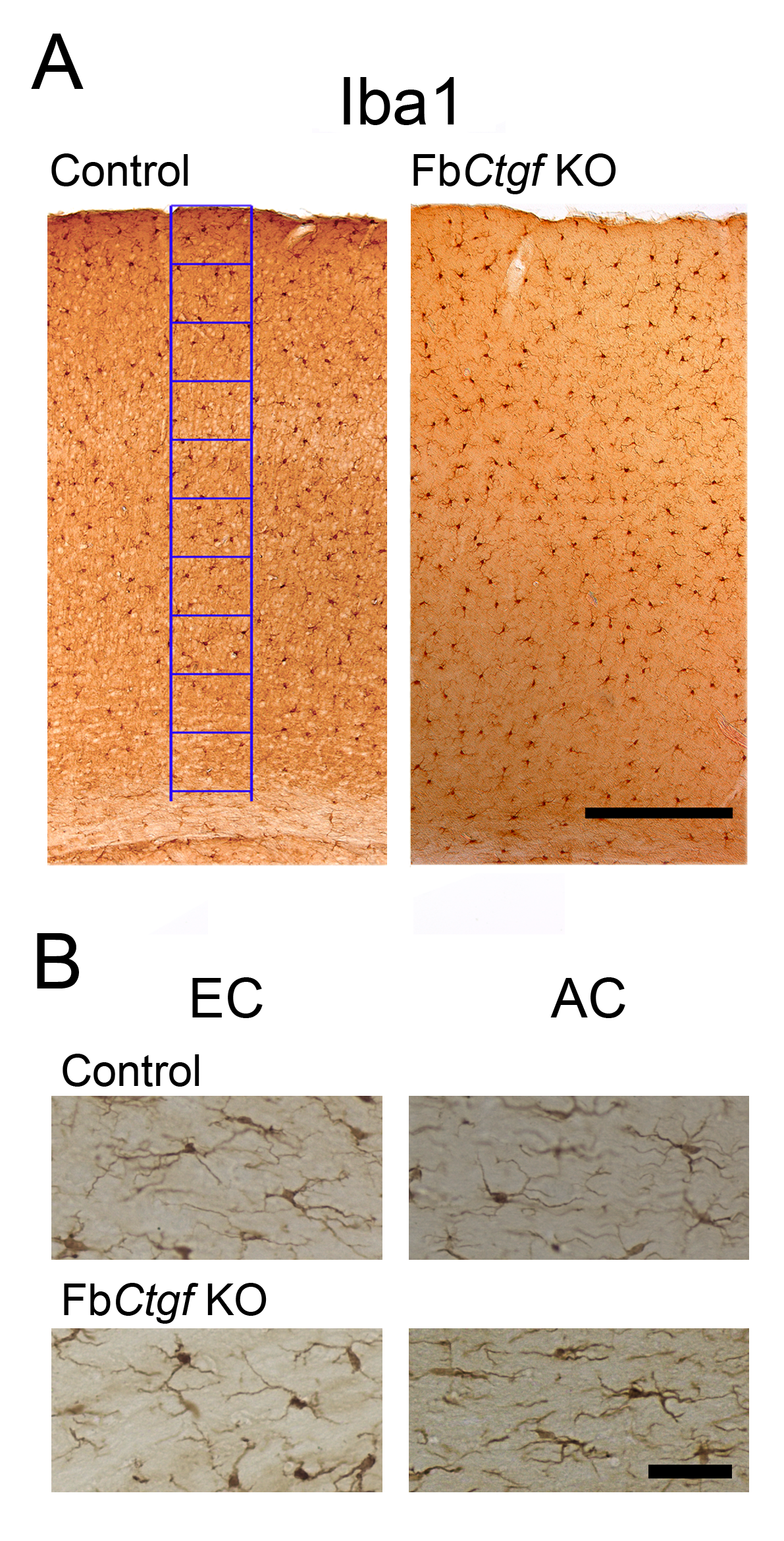

Supplement: Supplementary file 2 [file Image_2.TIF]

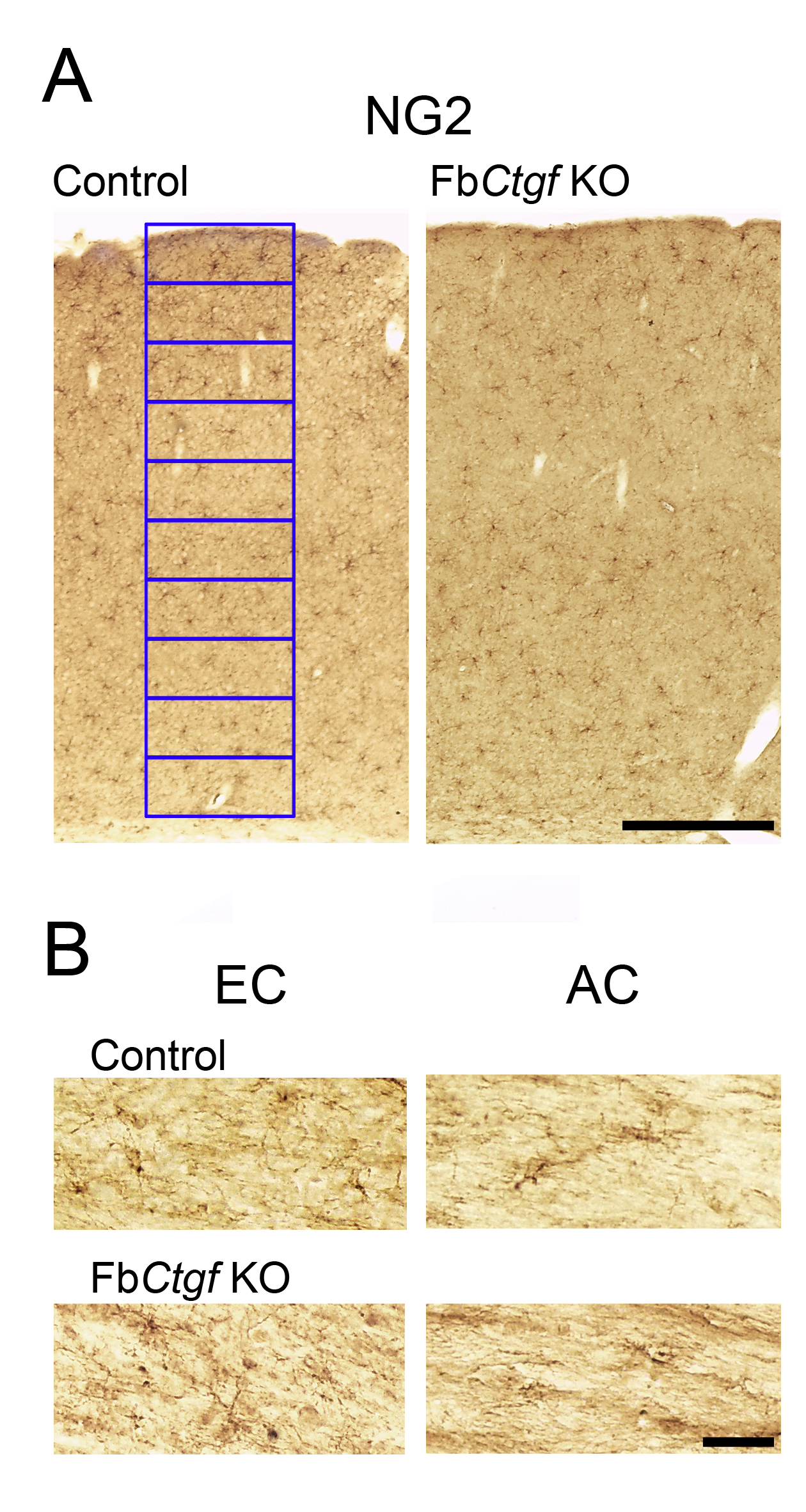

Supplement: Supplementary file 3 [file Image_3.TIF]

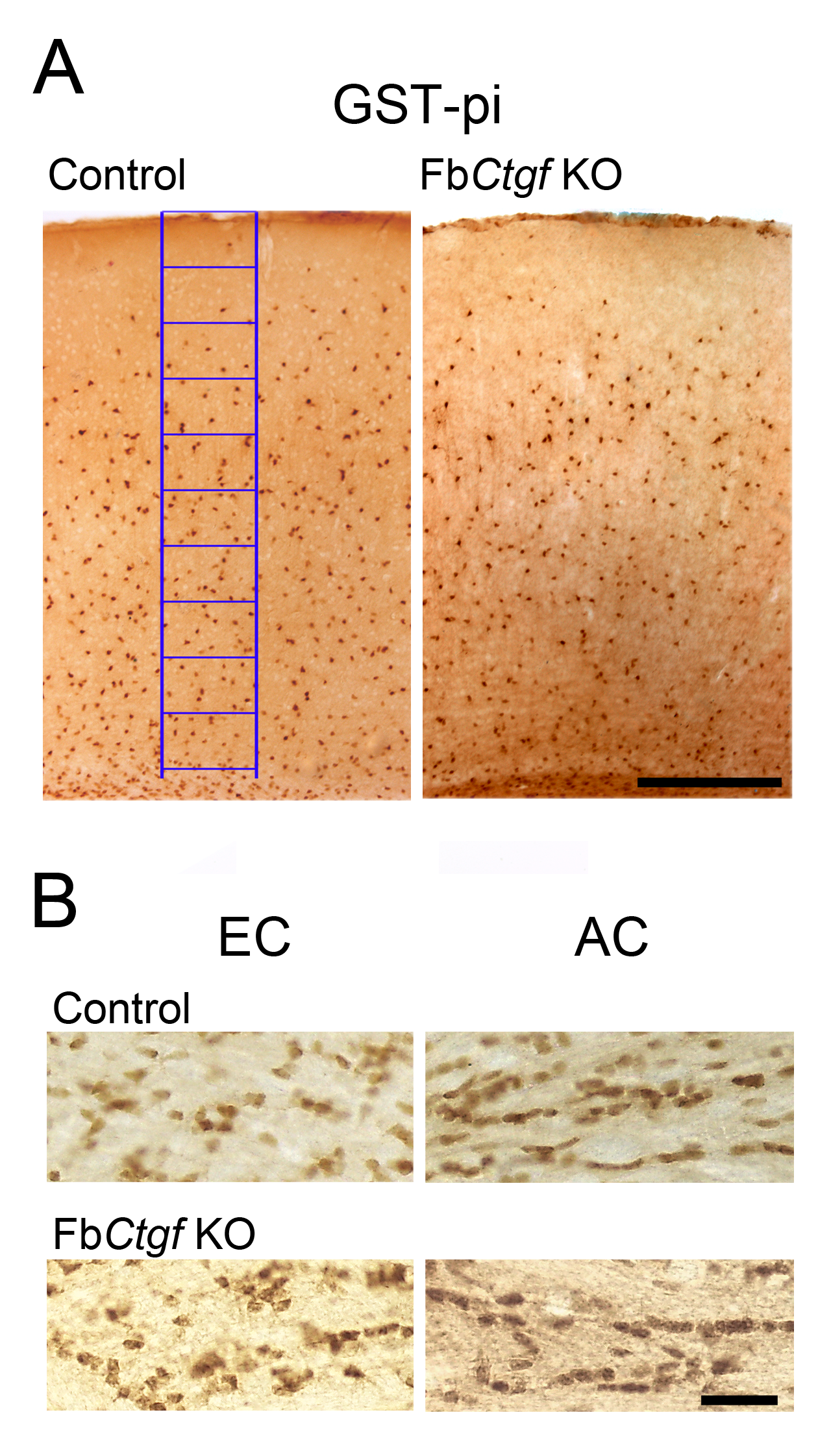

Supplement: Supplementary file 4 [file Image_4.TIF]
