# Supplementary material for: Genetic Elimination of Connective Tissue Growth Factor in the Forebrain Affects Subplate Neurons in the Cortex and Oligodendrocytes in the Underlying White Matter
Source: Front Neuroanat. 2019 Feb 20;13:16. doi: 10.3389/fnana.2019.00016 (PMC6391576; doi:10.3389/fnana.2019.00016)
Supplement: Supplementary file 5 [file Data_Sheet_1.docx]

**Supplementary Figure 1. Astrocytes in the brain**

Astrocytes were revealed by S100b immunohistochemistry in both control and Fb*Ctgf* KO mice. In the somatosensory cortex, the distribution of S100b-positive astrocytes was measured using 10 counting bins of 100 μm in width (blue bins) from the pia surface to the edge of the white matter (**A**). S100b-positive astrocytes were also present in the white matter, including the EC and AC (**B**). Scale bars are 200 μm in A and 50 μm in B.

**Supplementary Figure 2. Microglia in the brain**

Microglia were labeled by Iba1 immunohistochemistry in both control and Fb*Ctgf* KO mice. Iba1-positive microglia were evenly distributed in the somatosensory cortex (**A**). Microglia were also present in the white matter, including the EC and AC (**B**). Scale bars are 200 μm in A and 50 μm in B.

**Supplementary Figure 3. Immature oligodendrocytes in the brain**

Immature oligodendrocytes were labeled by NG2 immunohistochemistry in the somatosensory cortex (**A**) and white matter including EC and AC (**B**). Scale bars are 200 μm in A and 50 μm in B.

**Supplementary Figure 4. Mature oligodendrocytes in the brain**

Mature oligodendrocytes were revealed by GST-pi immunohistochemistry in the somatosensory cortex (**A**) and white matter structures including AC and EC (**B**). Scale bars are 200 μm in A and 50 μm in B.
